# Discovery of Adamantyl Heterocyclic Ketones as Potent 11β-Hydroxysteroid Dehydrogenase Type 1 Inhibitors

**DOI:** 10.1002/cmdc.201100144

**Published:** 2011-05-23

**Authors:** Xiangdong Su, Nigel Vicker, Mark P Thomas, Fabienne Pradaux-Caggiano, Heather Halem, Michael D Culler, Barry V L Potter

**Affiliations:** [a] Medicinal Chemistry, Department of Pharmacy and Pharmacology, University of BathClaverton Down, Bath BA2 7AY (UK), Fax: (+44) 1225-386-114; [b] IPSEN, Biomeasure Inc.27 Maple Street, Milford, MA 01757 (USA)

**Keywords:** 11β-HSD1 inhibitors, adamantyl ethanones, diabetes, hydroxysteroid dehydrogenases, metabolic syndrome

## Abstract

11β-Hydroxysteroid dehydrogenase type 1 (11β-HSD1) plays a key role in converting intracellular cortisone to physiologically active cortisol, which is implicated in the development of several phenotypes of metabolic syndrome. Inhibition of 11β-HSD1 activity with selective inhibitors has beneficial effects on various conditions, including diabetes, dyslipidemia and obesity, and therefore constitutes a promising strategy to discover novel therapies for metabolic and cardiovascular diseases. A series of novel adamantyl heterocyclic ketones provides potent and selective inhibitors of human 11β-HSD1. Lead compounds display low nanomolar inhibition against human and mouse 11β-HSD1 and are selective with no activity against 11β-HSD2 and 17β-HSD1. Selected potent 11β-HSD1 inhibitors show moderate metabolic stability upon incubation with human liver microsomes and weak inhibition of human CYP450 enzymes.

## Introduction

**Scheme 1:**
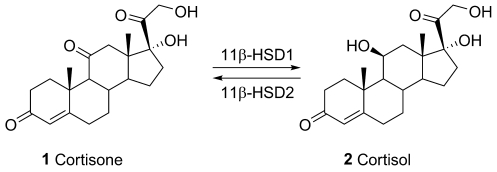
Interconversion of glucocorticoid hormones catalysed by 11β-HSDs.

11β-hydroxysteroid dehydrogenase isozymes (11β-HSDs) are microsomal enzymes from the short-chain dehydrogenase/reductase family that catalyse the intracellular conversion of physiologically active glucocorticoids and their inert 11-keto counterparts in specific tissues.[1, 2] The 11β-hydroxysteroid dehydrogenase type 1 (11β-HSD1), highly expressed in liver, adipose tissue and the central nervous system, acts as an NADPH-dependent reductase converting cortisone (**1**) in humans to the active glucocorticoid cortisol (**2**) (Scheme 1). The pre-receptor activation of cortisone mediated by 11β-HSD1 provides a mechanism for specific tissues to produce intracellular, nonadrenal cortisol, thereby locally intensifying the glucocorticoid action.[3] Conversely, the 11β-HSD2 isoform is exclusively NAD^+^ dependent and mainly expressed in mineralocorticoid target tissues, such as the kidney and colon. The 11β-HSD2 isoform catalyses the transformation of cortisol to inactive cortisone and reduces the local concentration of cortisol in specific tissues. This mechanism prevents cortisol occupation of mineralocorticoid receptors in the kidney, which may result in sodium retention, hypokalaemia and hypertension.[4–6]

The tissue-specific regulation of glucocorticoid by 11β-HSDs is of particular physiological importance, as cortisol in humans plays essential roles in the regulation of carbohydrate, lipid and bone metabolism, modulation of inflammatory responses, brain function, and stress. Recent investigations have shown that excessive glucocorticoid action is, in many respects, associated with insulin and leptin resistance, leading to the development of type 2 diabetes, obesity and other metabolic and cardiovascular disorders, the major underlying causes of metabolic syndrome.[7–10] Glucocorticoid receptor (GR) activation stimulates hepatic glucose production, antagonises insulin secretion from pancreatic β-cells and insulin-mediated glucose uptake in peripheral tissues.[11–14] GR activation also promotes lipolysis and fatty acid mobilisation.[15]

In many aspects, metabolic syndrome shares similar characteristics with symptoms of Cushing’s syndrome, a systemic glucocorticoid excess condition, characterised by insulin resistance, high adiposity, dyslipidemia, and hypertension.[16] These metabolic abnormities in Cushing’s syndrome can be improved to a certain degree by reducing the excessive glucocorticoid action through surgery or glucocorticoid receptor antagonist treatment.[17–19] The similarities observed between phenotypes of Cushing′s syndrome and metabolic syndrome suggest the possibility of treating the individual indications of metabolic syndrome by glucocorticoid activity suppression.[20] GR signalling depends not only on the circulating glucocorticoid levels, but also on the pre-receptor activation of glucocorticoid within cells. Since systemic glucocorticoid levels are generally normal in patients with common forms of obesity or overweight type 2 diabetics,[21] it is speculated that the intracellular glucocorticoid concentration, regulated by 11β-HSDs, is responsible for the metabolic abnormalities. Studies show that 11β-HSD1 expression is elevated in adipose tissue of obese subjects suggesting the possibility of tissue-specific local glucocorticoid excess.[22, 23]

The correlation between 11β-HSD1 activity, obesity and diabetes has also been validated with genetically modified rodent models. Transgenic mice with overexpression of 11β-HSD1 in fat tissue specifically developed symptoms of insulin-resistant diabetes, hyperlipidemia and visceral obesity.[24, 25] In contrast, studies with 11β-HSD1 knock-out mice demonstrated that these animals resist stress-induced hyperglycaemia, diet-induced obesity, and have decreased cholesterol and triglyceride levels.[26] Similarly, overexpression of 11β-HSD2 in transgenic mice also results in increased insulin sensitivity, glucose tolerance, and resistance to body-weight gain on a high-fat diet.[27] 11β-HSD1 activity suppression in animal models with selective inhibitors was found to provide beneficial effects on various indications of metabolic syndrome.[28–30] Moreover, clinical studies suggest that inhibition of 11β-HSD1 with carbenoxolone, a nonselective inhibitor, increased hepatic insulin sensitivity and decreased glucose production.[31, 32]

All this evidence supports the idea of treating type 2 diabetes, obesity and other metabolic abnormalities through selective inhibition of 11β-HSD1 activity with small-molecule inhibitors.[33] This approach has attracted considerable interest in the pharmaceutical industry over the last decade,[34–37] which has led to the discovery of numerous types of potent, selective 11β-HSD1 inhibitors.[37–42] Results from a positive proof-of-concept clinical study of the potent 11β-HSD1 inhibitor INCB013739 developed by Incyte provided substantial evidence that the inhibition of 11β-HSD1 can be a viable treatment of type 2 diabetes. It was reported that INCB013739 treatment of type 2 diabetes mellitus patients who failed on metformin monotherapy could significantly improve hepatic and peripheral insulin sensitivity and reduce haemoglobin A1c and fasting plasma glucose. In patients with hyperlipidaemia or hypertriglyceridaemia, treatment with INCB013739 also lowers triglyceride and cholesterol levels.[43, 44]

**Figure 1:**
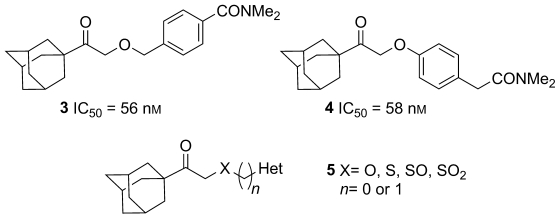
Potent inhibitors of 11β-HSD1 (**3**, **4**) and the general structure (**5**) for optimisation. For full structures, see Tables 1–6.

To discover novel, clinically useful inhibitors of 11β-HSD1, it is important to have an array of structural types of inhibitor, as the physicochemical properties of the compounds will determine tissue distribution, hypothalamic–pituitary–adrenal axis effects, and, ultimately, clinical utility. Previously, we reported that some adamantyl ethanone derivatives possess high inhibitory activity on the cellular 11β-HSD1 enzyme.[45] Compounds **3** and **4** exhibit potent inhibition of 11β-HSD1 with IC_50_ values of approximately 60 nm. To further improve potency, pharmacokinetic properties and physicochemical properties, we performed optimisation on this series of compound using structure-based design.[46] Compounds containing an adamantyl group linked to a heterocyclic unit through a multiatom linker with a carbonyl group attached to the adamantane, as illustrated by general structure **5**, were synthesised (Figure 1). These target compounds were screened for their inhibitory activity against human 11β-HSD1 in a HEK293 cell-based assay. Selected potent compounds were tested for inhibitory activity against mouse 11β-HSD1. Their selectivities over 11β-HSD2 and 17β-HSD1 were also examined.

## Results and Discussion

### Chemistry

**Scheme 2:**
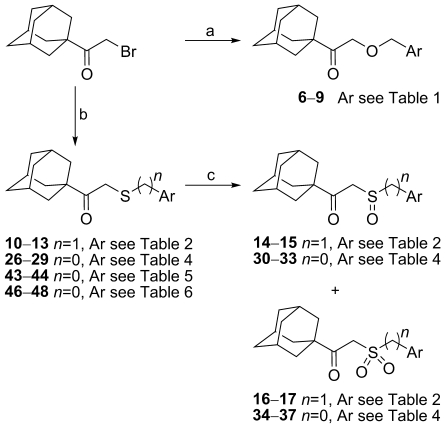
Synthesis of the adamantyl ethanone compounds. *Reagents and conditions*: a) ArCH_2_OH, NaH, THF, 0 °C; b) ArCH_2_SH, Et_3_N, CH_3_CN, RT; c) *m*-CPBA, CH_2_Cl_2_, −10→0 °C.

The adamantyl derivatives with an ethanone ether linker (**6**–**9**) were generated by a nucleophilic coupling reaction between the corresponding aryl methyl alcohols and 1-adamantyl bromomethyl ketone under basic conditions (Scheme 2). Most of the target compounds with a sulfur linker can be prepared by a coupling reaction of 1-adamantyl bromomethyl ketone with the corresponding commercially available mercaptan in the presence of triethylamine in acetonitrile. Further oxidation of these compounds with *meta*-chloroperoxybenzoic acid (*m-*CPBA) at −10 °C to 0 °C generally produced both the sulfoxide (**14, 15, 30**–**33**) and sulfone (**16, 17, 34**–**37**) derivatives (Scheme 2), which could be separated by flash column chromatography.

**Scheme 3:**
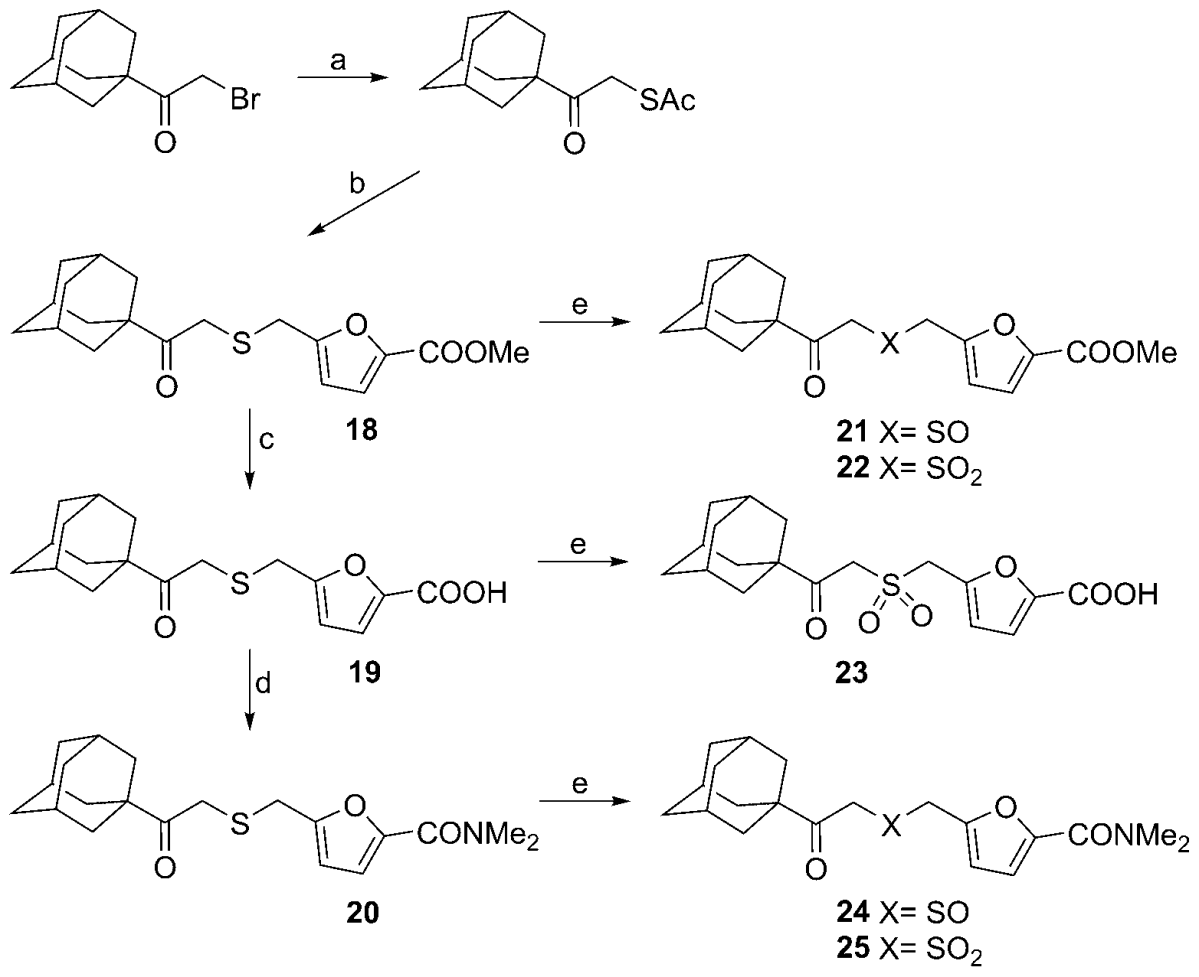
Synthesis of furanyl adamantyl ethanone derivatives (**18**–**25**). *Reagents and conditions*: a) AcSK, CH_3_CN, RT; b) 1. 1 n NaOH, acetone; 2. methyl-5-(chloromethyl)furan-2-carboxylate, RT; c) KOH, CH_3_OH, RT; d) NHMe_2_, EDCI, DMAP, Et_3_N, CH_2_Cl_2_, RT; e) *m*-CPBA, CH_2_Cl_2_, −10→0 °C.

Synthesis of the furanylmethylthio 1-adamantylethanone derivative **18** was performed through a nucleophilic substitution, followed by a coupling reaction of ethanthioate with methyl 5-(chloromethyl)furan-2-carboxylate as illustrated in Scheme 3. Hydrolysis of the methyl ester and subsequent amide formation gave compounds **19** and **20**. Their corresponding sulfoxide (**21**, **24**) and sulfone derivatives (**22**, **23** and **25**) were synthesised by oxidation with *m*-CPBA (Scheme 3). No sulfoxide product was obtained from the oxidation of **19** under these conditions.

**Scheme 4:**
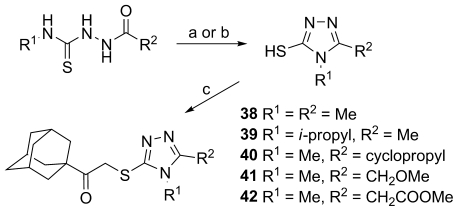
Synthesis of 1,2,4-triazole derivatives (**38**–**42**). *Reagents and conditions*: a) 2 n NaOH; b) 1. 2 n NaOH; 2. HCl, CH_3_OH; c) 1-adamantyl bromomethyl ketone, Et_3_N.

Target compounds **38**–**42** were synthesised from substituted 4*H*-1,2,4-triazole-3-thiol intermediates, which were obtained through the cyclisation of substituted hydrazinecarbothioamide under basic conditions (Scheme 4).

**Scheme 5:**
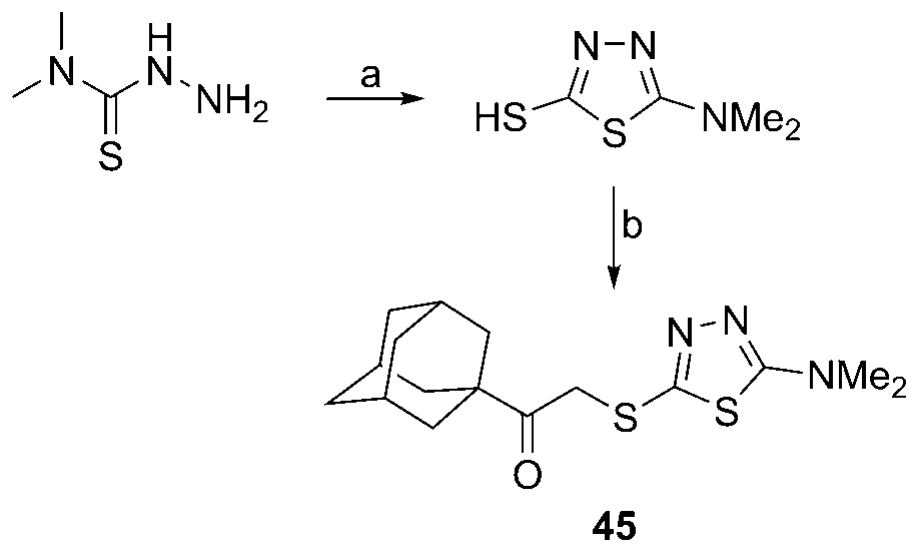
Synthesis of 1,3,4-thiadiazole derivative (**45**). *Reagents and conditions*: a) CS_2_, Et_3_N, DMF; b) 1-adamantyl bromomethyl ketone, Et_3_N (54 % yield).

Most of the designed 1,3,4-thiadiazole derivatives can be made from the commercially available substituted (1,3,4-thiadiazol-2-yl)methanethiol via a coupling reaction with 1-adamantyl bromomethyl ketone. The 5-*N,N*-dimethylamine substituted compound (**45**) was prepared by cyclisation of *N,N*-dimethylhydrazinecarbothioamide with carbon disulfide, followed by a coupling reaction as shown in Scheme 5.

### Structure–activity relationships (SAR)

The target compounds were examined for their inhibition against 11β-HSD1 on an HEK293 cell line transfected with the human HSD11B1 gene. Our previous study found that adamantyl ethanone derivatives exhibited 11β-HSD1 inhibitory activity with lead compounds showing IC_50_ values in the range of 50–60 nm.[45] Compounds **3** and **4**, with a substituted benzyl or phenyl group, respectively, attached to the adamantyl ethanone through an ether linker, demonstrate high potency, good selectivity over 11β-HSD2, and moderate metabolic stability in human liver microsomes.[45] Therefore, the adamantyl ethanone moiety represents a suitable template for further optimisation.

Replacing the phenyl group of compound **3** with 2-thiophene generated compound **6**, which shows moderate activity (IC_50_=410 nm). Moving the sulfur to the 3-position only increases the activity slightly (**7**, IC_50_=280 nm). However, the introduction of a 2-thiazole ring results in a tenfold improvement in inhibitory activity, generating the highly potent compound **8** with an IC_50_ value of 41 nm. The improvement in activity may be due to the added interaction of the nitrogen atom with the protein in the binding site or the change in molecular physicochemical properties. When a methyl group was added to the 5-position of the thiazole ring, the activity reduces slightly (**9**, Table 1).

**Table 1:** In vitro inhibition of compounds **6**–**9**

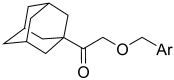		
Compd	Ar[a]	IC_50_[b] [nm]
**6**	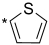	410
**7**	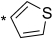	280
**8**	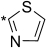	41
**9**	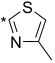	71

[a] Point of attachment indicated by *.

[b] The IC_50_ values are reported as the mean of three measurements with variance less than 20 %.

**Figure 2:**
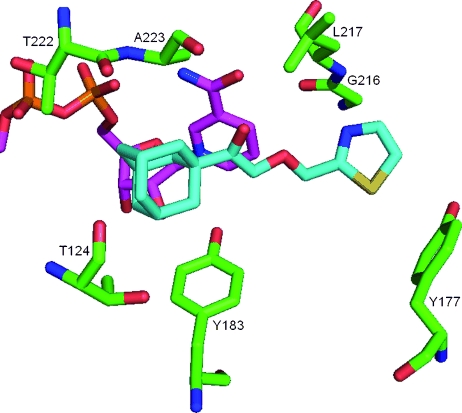
Docking pose of compound **8** (cyan) with cofactor (purple) and active site residues (green).

To investigate the possible binding mode of the inhibitor, compound **8** was docked into the published X-ray crystal structure of 11β-HSD1 (PDB: 2ILT[47]) using the GOLD docking program (version 4.1). The best docking solution predicts that the adamantyl group will pack nicely between the nicotinamide ring of the cofactor and Y183, and the carbonyl is able to stack with the nicotinamide amide. The thiazole ring is predicted to have an edge-on interaction with Y177 (∼4 Å) (Figure 2).

We also evaluated the effect of replacing the oxygen linker with a sulfur atom in the same core structure (Table 2). Compared with **6**, the activity of compound **10** increases by nearly threefold with an IC_50_ value of 143 nm, suggesting that the sulfur linker may enable more favourable interactions with the protein. Similarly, compound **11** with the furan-2-yl group is equally potent. However, this effect was not observed in compounds with a thiazolyl group in the aromatic region. Compounds **12** (IC_50_=244 nm) and **13** (IC_50_=327 nm) exhibit reduced activity compared to their oxygen linker countparts **8** and **9**, respectively, indicating that the linker change may alter the binding position of the thiazolyl ring. Interestingly, we found that the change of the sulfide linker to a sulfoxide greatly raises the inhibitory activity to under 50 nm for both thiophenyl and furanyl derivatives (**14**, **15**), suggesting that the oxygen on the sulfur possibly forms further interactions with the enzyme or alters the geometry of the molecule placing the adamantyl and/or the aromatic ring in a position to gain further binding interactions in the active site. Compound **16**, with a sulfone linker, exhibits similar potency compared with the sulfide analogue **10**, but a fourfold weaker potency compared with the sulfoxide compound **14**. However, compound **17** with a furanyl ring maintains the same potency (IC_50_=36 nm) as its sulfoxide analogue **15**. A docking study with **17** found that there is a possible hydrogen-bond interaction from S170 to the carbonyl and from Y183 to either the carbonyl or one of the SO_2_ oxygen atoms.

**Table 2:** In vitro inhibition of compounds **10**–**17**

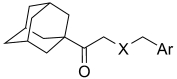			
Compd	X	Ar[a]	IC_50_[b] [nm]
**10**	S	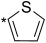	143
**11**	S	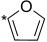	163
**12**	S	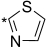	244
**13**	S	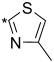	327
**14**	SO	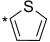	46
**15**	SO	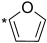	41
**16**	SO_2_	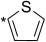	202
**17**	SO_2_	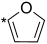	36

[a] Point of attachment indicated by *.

[b] The IC_50_ values are reported as the mean of three measurements with variance less than 20 %.

Based on the positive results from the adamantyl ethanone furanyl series, the possibility of altering the substitution on the furan ring was explored (Table 3), as our previous work indicated that a hydrogen-bonding group on the aromatic ring might potentially improve the inhibition of 11β-HSD1.[45] Surprisingly, the 5-methyl ester derivative **18** shows reduced activity (IC_50_=264 nm) compared with the unsubstituted compound **11** (IC_50_=163 nm). Although the sulfoxide linker analogue **21** regains the activity by 2.6-fold, with an IC_50_ value of 102 nm, it is still less potent in comparison with the unsubstituted sulfoxide (**15**, IC_50_=41 nm). The sulfone-linked compound **22** exhibits only 15 % of the potency of the unsubstituted compound **17**. Two compounds (**19** and **23**) with a free carboxylic acid on the 5-position both gave poor activity, possibly due to their low permeability when tested on the HEK293 cell line. Compound **20** (IC_50_=153 nm), with the *N*,*N*-dimethylcarboxamide substitution, is equally potent as the unsubstituted compound **11**; however, alteration of the linker to sulfoxide or sulfone (**24** and **25**) does not improve the inhibitory activity in the same way as that observed in the unsubstituted series (**15** and **17**).

**Table 3:** In vitro inhibition of compounds **18**–**25**

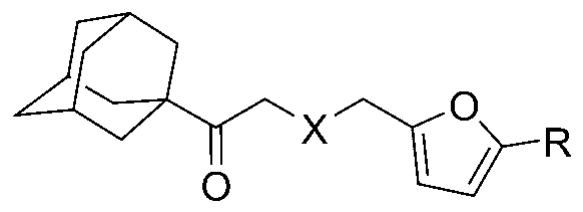			
Compd	X	R	IC_50_[a] [nm]
**18**	S	COOMe	264
**19**	S	COOH	67 %[b]
**20**	S	CONMe_2_	153
**21**	SO	COOMe	102
**22**	SO_2_	COOMe	239
**23**	SO_2_	COOH	4 %[b]
**24**	SO	CONMe_2_	144
**25**	SO_2_	CONMe_2_	122

[a] The IC_50_ values are reported as the mean of three measurements with variance less than 20 %.

[b] Percent inhibition measured at a concentration of 1 μm and reported as the mean value of three experiments with variance <20 %.

From the above SAR data, we found that the core structure of adamantyl ethanone linked to methyl 2-thiophenyl or methyl 2-furanyl moieties through a sulfoxide or sulfone group normally generates potent inhibitors of 11β-HSD1. Compounds **14**, **15** and **17**, with IC_50_ values below 50 nm, are the most potent inhibitors from this new series (Table 2 and Table 3).

**Figure 3:**
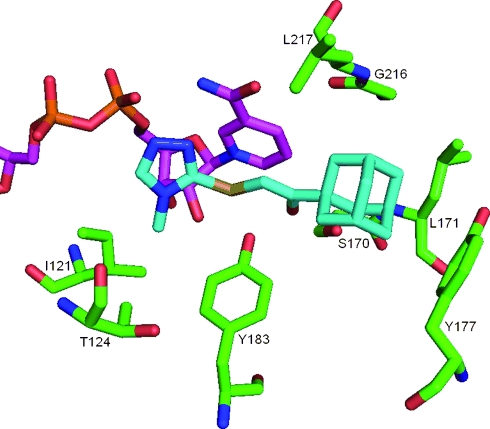
Docking solution of compound **28** (cyan) with cofactor (purple) and active site residues (green).

To investigate the SAR of adamantyl ethanone derivatives with the heterocyclic ring attached directly to a sulfur linker, we synthesised four series of compound containing different 5-membered ring systems and evaluated their inhibition (Table 4). The thiophenyl derivative **26** (IC_50_=101 nm) shows activity similar to analogue **10**, which has an extra methylene unit between the sulfur and aromatic ring. Replacing the thiophene ring with 1-methyl-1*H*-imidazole or 5-methyl-1,3,4-thiadiazole gave two compounds with slightly improved potency (**27**: IC_50_=93 nm; **29**: IC_50_=61 nm). Interestingly, when a 4-methyl-4*H*-1,2,4-triazole moiety was introduced, the most potent compound **28**, with an IC_50_ value of 19 nm, was identified. The 4*H*-1,2,4-triazole core structure can be found in many potent 11β-HSD1 inhibitors reported previously.[48–51] The dramatic increase in activity could be the result of a network of interactions with the triazole ring. A docking study with compound **28** found that the highest docking score solution poses the triazole ring close to the cofactor with the triazole methyl group fitting in a pocket formed by I121, T124 and Y183. The carbonyl group is predicted to form hydrogen bonds to Y183 and S170 (both ∼2.5 Å). The adamantyl group is predicted to fit tightly in a hydrophobic pocket formed by L171, Y177 and L217 (Figure 3).

**Table 4:** In vitro activity of compounds **26**–**37**

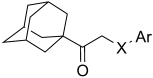			
Compd	X	Ar[a]	IC_50_[b] [nm]
**26**	S	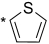	101
**27**	S	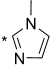	93
**28**	S	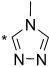	19
**29**	S	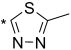	61
**30**	SO	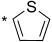	59
**31**	SO	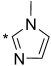	48
**32**	SO	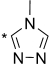	27
**33**	SO	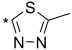	26
**34**	SO_2_	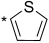	667
**35**	SO_2_	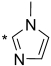	62
**36**	SO_2_	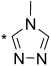	95
**37**	SO_2_	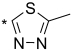	31

[a] Point of attachment indicated by *.

[b] The IC_50_ values are reported as the mean of three measurements with variance less than 20 %.

All compounds with a sulfoxide linker (**30**–**33**) are highly potent. Among them, the 4*H*-1,2,4-triazole derivative **32** and the 5-methyl-1,3,4-thiadiazole derivative **33** are the most potent, with IC_50_ values of 27 nm and 26 nm, respectively. In comparison with their sulfide linker analogues, compounds **30**, **31** and **33** all exhibit nearly double the potency. This observation is in agreement with that seen in the previous series (Table 2), indicating that the sulfoxide linkers may not only change the geometry of the molecule but may also act as hydrogen-bond acceptors with amino acid residues in the active site of 11β-HSD1.

The sulfone version of this series displays mixed results. The thiophenyl derivative **34**, the least potent in this series, loses 11-fold activity compared with the sulfoxide analogue **30**. Similarly, triazole derivative **36** (IC_50_=95 nm) has an activity 3.5-fold lower than the sulfoxide analogue **32**, and fivefold lower than the sulfide analogue **28**. However, compound **37** (IC_50_=31 nm) still retains the same level of potency as the compound with a sulfoxide linker (**33**, Table 4). As one of the most potent compounds with a sulfone linker, this compound is of particular interest for further investigation.

Having found that the 4*H*-1,2,4-triazole and 5-methyl-1,3,4-thiadiazole derivatives display significant inhibition against 11β-HSD1, we investigated the SAR for variation of substituents with different size and electronic properties on the aromatic ring (Tables 5 and 6). The 5-methyl substituent on the 4*H*-1,2,4-triazole ring gives a compound with relatively high potency (**38**, IC_50_=36 nm; Table 5); however, it is approximately twofold less active than the unsubstituted derivative **28**. Substitution with a hydrophobic cyclopropyl group (**40**, IC_50_=129 nm) results in a further decrease in activity by a further 3.6-fold, suggesting that the size of the substituent permitted in the aromatic region could be limited. The 5-thiophenyl-substituted compound **44** exhibits very low activity with an IC_50_ value of 5871 nm. This dramatic loss in activity may be due to an alteration of the binding mode caused by the biaryl unit or the physiochemical properties of the molecule. The introduction of a group with hydrogen bonding capacity at the 5-position keeps the IC_50_ value in the range of 100–150 nm (**41** and **42**, Table 5); however, these compounds are three- to fourfold less active than the 5-methyl analogue **38** and much less active than the unsubstituted compound **28**. Replacement of the methyl group of compound **38** with an *iso*-propyl group leads to a 3.8-fold loss of activity (**39**, IC_50_=137 nm), suggesting that the bulkier groups are not favoured in that region. This observation is in agreement with the result for compound **43** (IC_50_=1857 nm), with cyclopropyl substituents at both the 1- and 5-positions.

**Table 5:** In vitro activity of 1,2,4-triazole derivatives

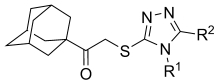			
Compd	R^1^	R^2^	IC_50_[a] [nm]
**38**	Me	Me	36
**39**	*i*-propyl	Me	137
**40**	Me	cyclopropyl	129
**41**	Me	CH_2_OMe	153
**42**	Me	CH_2_COOMe	97
**43**	cyclopropyl	cyclopropyl	1857
**44**	Me	2-thienyl	5871

[a] The IC_50_ values are reported as the mean of three measurements with variance less than 20 %.

**Table 6:** In vitro activity of 1,3,4-thiadiazole derivatives **45**–**52**

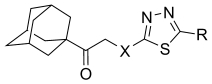			
Compd	X	R	IC_50_[b] [nm]
**45**	S	NMe_2_	4005
**46**	S	SMe	306
**47**	S	SEt	68 %[b]
**48**	S	NH_2_	71
**49**	SO_2_	NH_2_	1787
**50**	S	NHAc	848
**51**	SO	NHAc	333
**52**	SO_2_	NHAc	309

[a] The IC_50_ values are reported as the mean of three measurements with variance less than 20 %.

[b] Percent inhibition measured at a concentration of 1 μm and reported as the mean value of three experiments with variance <20 %.

A series of 1,3,4-thiadiazole derivatives with varied substituents in the 5-position was studied for inhibitory activity (Table 6). The methylthio- or ethylthio-substituted compounds (**46** and **47**) only exhibit moderate inhibition of 11β-HSD1. Compound **46**, with an IC_50_ value of 306 nm, shows 20 % of the activity of the methyl-substituted **29**. The 5-*N*,*N*-dimethylamino substituent also has a strongly negative effect on the activity (**45**, IC_50_=4005 nm), suggesting the possibility of limited steric and/or electronic requirements in a confined region.

It is interesting to note that the amino-substituted compound (**48**), with an IC_50_ value of 71 nm, shows nearly the same level of potency as the methyl-substituted compound **29**. The benefit of having an amino group on the 1,3,4-thiadiazole ring is that it can provide a handle for further optimisation. A docking study suggested the possibility of the thiadiazole amino group of **48** forming hydrogen bonds with NADP phosphate (3.4 Å), and the carbonyl group interacting with Y183 (2.8 Å) and S170 (2.5 Å) also through hydrogen bonds. It was surprising to see the sulfone-linked analogue **49** exhibiting only modest activity with an IC_50_ value of 1787 nm. The acetamide substituent (**50**, IC_50_=848 nm) reduces the activity of **48** by almost 12-fold, providing further proof that the size of the substituent is crucial to the activity. Although oxidation of the sulfide linker of **50** to sulfoxide or sulfone raised the inhibitory activity by about 2.5-fold, compounds **51** (IC_50_=333 nm) and **52** (IC_50_=309 nm) are still far less potent than their methyl-substituted analogues **33** and **37**.

Having identified ten potent compounds, with IC_50_ values below 50 nm, from these adamantyl ethanone derivatives, we examined their inhibition against mouse 11β-HSD1, since a mouse model will most likely be used in vivo. Compounds **8**, **14**, **15**, **33** and **37** all exhibit similar levels of potency against the mouse and human enzymes, which makes them suitable for further evaluation in mouse models. Although compounds **17**, **28**, **31** and **38** are several fold less active on the mouse enzyme, they still show relatively strong activity, especially compound **28** (human: IC_50_=19 nm, mouse: IC_50_=61 nm; Table 7). Compounds **17**, **28**, **31** and **38** were also tested for their inhibition against human 11β-HSD2 and 17β-HSD1. At a concentration of 10 μm, these compounds are all inactive (data not shown) and therefore are regarded as highly selective 11β-HSD1 inhibitors.

**Table 7:** Inhibition of human and mouse 11β-HSD1

Compd	IC_50_[a] [nm]	
	human	mouse
**8**	41	18
**14**	46	20
**15**	41	38
**17**	36	176
**28**	19	61
**31**	48	121
**32**	27	3084
**33**	26	44
**37**	31	57
**38**	36	248

[a] The IC_50_ values are reported as the mean of three measurements with variance less than 20 %.

**Scheme 6:**
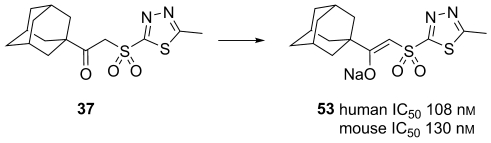
Potent compound **53** with improved solubility.

To improve the solubility of an adamantyl ethanone derivative, compound **37** was transformed into the enolate sodium salt (**53**) (Scheme 6), which exhibits enhanced water solubility (>4 mg mL^−1^) in a Na_2_HPO_4_ buffer solution with or without 5 % *N*,*N*-dimethylacetamide (DMA) as a cosolvent. Compound **53** displays relatively high potency against 11β-HSD1 with an IC_50_ value of 108 nm.

### Permeability, metabolic stability and CYP450 inhibition studies

The membrane permeability of two selected compounds (**14** and **31**) was determined using the Caco-2 cell model. The apparent permeability coefficient (*P*_app_) of each drug from the apical to basolateral side was measured at a concentration of 20 μm. The results indicate that both compounds possess high permeability (**14**: *P*_app_ (A>B)=2.2×10^−5^; **31**: *P*_app_ (A>B)=4.4×10^−5^), which supports the fact that these molecules are of a lipophilic nature.

Potent inhibitors **15, 17, 28** and **37** were evaluated for their metabolic stability in human liver microsomes (Table 8). Compounds **15** and **37** both suffer from rapid metabolism under the test conditions, with a half-life of only 30–40 min and a high clearance rate. However, triazole derivative **28** shows improved metabolic stability with a half-life of more than 1 h and an intrinsic clearance of ∼9 μL min^−1^ mg^−1^. More interestingly, compound **17** is relatively stable under the human liver microsomes incubation conditions, displaying a half-life of more than 4 h and a clearance rate below 3 μL min^−1^ mg^−1^. This clearly indicates that metabolic stability suitable for a compound to be dosed in vivo may be achieved with this structural template.

**Table 8:** Metabolism studies in human liver microsomes.[a]

Compd	*t*_1/2_ [min]	CL_int_ [μL min^−1^ mg^−1^]
**15**	39	17.6
**17**	257	2.9
**28**	68	8.7
**37**	34	19

[a] The parent compound is incubated at 37 °C with human liver microsomes in presence of the cofactor NADPH for 40 min. Disappearance of the parent compound is monitored using a HPLC system. Half-life (*t*_1/2_) and intrinsic clearance (CL_int_) values were calculated accordingly.[52] Data reported as the mean value of two experiments.

To examine the possibility of these compounds interfering with the metabolism of other drugs, compounds **14**, **17, 28** and **37** were tested for their inhibition of key human cytochrome P450 enzymes: 1A2, 2C9, 2C19, 2D6, 3A4-BFC and 3A4-BQ (Table 9). Compound **14** shows very weak activity against 1A2, 2C19, 3A4-BFC and 3A4-BQ. Compound **28** displays moderate inhibition of 2C19, with an IC_50_ value of 3.6 μm, and compound **37** inhibits 3A4-BFC with an IC_50_ value of 6.3 μm. Compound **17**, although showing no inhibition for 1A2 and 2D6, exhibits inhibition at a concentration of 10 μm for 2C9 (52 %), 2C19 (92 %), 3A4-BFC (86 %) and 3A4-BQ (52 %).

**Table 9:** Inhibition of human cytochrome P450 enzymes by selected analogues

CYP450	Compd **14**	Compd **17**[a]	Compd **28**	Compd **37**
1A2	33 μm	0	21 μm	>100 μm
2C9	ND	52 %[a]	ND	11 μm
2C19	30 μm	92 %[a]	3.6 μm	17 μm
2D6	ND	0	ND	>100 μm
3A4-BFC[b]	>100 μm	86 %[a]	>100 μm	6.3 μm
3A4-BQ[c]	12 μm	52 %[a]	62 μm	30 μm

[a] Percent inhibition measured at a concentration of 10 μm.

[b] BFC=7-benzyloxy-trifluoromethylcoumarin

[c] BQ=7-hydroxy-quinoline ND: not determined. Data reported as the mean value of two measurements.

## Conclusions

In summary, we have identified several new series of adamantyl ethanone heterocyclic derivatives as novel inhibitors of human and mouse 11β-HSD1. The ten most potent compounds (Table 7) display selective high inhibitory activity against 11β-HSD1, with IC_50_ values below 50 nm, when evaluated on a stably transfected HEK293 cell line. It was found that an adamantyl ethanone motif tethered to a 5-membered aromatic heterocycle through an oxygen, sulfur, sulfoxide or sulfone linker provides a suitable pharmacophore for inhibition of 11β-HSD1. The further optimisation of the previously reported compounds **3** and **4** lead to a potent inhibitor with approximately a threefold improvement in activity. Compound **28** (11β-HSD1, human IC_50_=19 nm, mouse IC_50_=61 nm) displays reasonable metabolic stability under human liver microsomes incubation conditions with a half-life of 68 min and very weak inhibition of key human CYP450 enzymes. This compound, in particular, is regarded as a candidate for further preclinical investigation.

## Experimental Section

**11β-HSD1 inhibition cellular assay using a scintillation proximity assay (SPA) protocol**:[53] Wild type HEK293 cells lack endogenous 11β-HSD1 activity and this cell line has been shown to be a suitable system for evaluating 11β-HSD1 activity after being transfected with the plasmid for expression of 11β-HSD1 or 11β-HSD2. The enzyme activity was determined by measuring the amount of tritiated product by using a scintillation proximity assay (SPA). The high-throughput cell-based assays were conducted on the HEK293 cell line stably transfected with the HSD11B1 gene using modified literature protocols. Cells were incubated in 96-well micro-plates in the presence of tritiated substrate and the assay plates contained internal high and low controls to allow calculation of percentage inhibition. Each well of a 96-well culture plate was seeded with HEK293/HSD11B1 cells in 100 μL medium. When the cells were 80 % confluent, the medium was removed from each well then 100 μL of fresh, serum-free, medium containing [^3^H]cortisone and test compound in 1 % DMSO was added to each well. The control wells were also dispensed. The high control wells did not contain compound, while low controls did not contain cells. The plate was incubated at 37 °C for the required time period, after which, 50 μL of media was removed from each well and transferred to a microplate containing 100 μL of a pre-incubated mixture of anti-cortisol antibody and SPA bead. The mixture was incubated with gentle shaking until equilibrium was reached, before transferring to a scintillation counter to establish the enzyme activity in each sample.

**Docking study procedure**: Selected ligands were docked into the human 11β-HSD1 protein X-ray crystal structure (PDB: 2ILT[47]) using the GOLD docking program (version 4.1) with default settings in the presence of the cofactor. The binding site was defined as a sphere of 10 Å radius around the centroid of the ligand in the 2ILT structure. Each ligand was docked 25 times. The GOLDscore scoring function was used to rank the ligands in order of fitness to generate the best poses of the ligand in the active site.

**General methods for synthesis**: 1-Adamantyl bromomethyl ketone was obtained from Aldrich Chemical Co. (Gillingham, UK). All other chemicals were purchased from either Aldrich Chemical Co. or Alfa Aesar (Heysham, UK). All organic solvents of AR grade were supplied by Fisher Scientific (Loughborough, UK). Melting points were determined using a Stanford Research Systems Optimelt MPA100 and are uncorrected. Compounds in solid form were crystallised from CH_2_Cl_2_/EtOAc. Thin layer chromatography (TLC) was performed on pre-coated aluminium plates (Merck, silica gel 60 F_254_). Products were visualised by UV irradiation at 254 nm and staining with 5 % *w*/*v* molybdophosphoric acid in EtOH, followed by heating. Flash column chromatography was performed on pre-packed columns (RediSep *R*_f_) and gradient elution (solvents indicated in text) on the Combiflash RF system (Teledyne Isco). ^1^H NMR spectra were recorded on a Jeol Delta 270 mhz spectrometer. Chemical shifts (δ) are reported in parts per million (ppm) relative to tetramethylsilane (TMS) as an internal standard. LC/MS spectra were recorded on a Waters 2790 machine with Waters “Symmetry” C18 column (packing: 3.5 μm, 4.6 mm × 75 mm) eluting with 10 % H_2_O/CH_3_OH (1 mL min^−1^), and detected with a ZQ MicroMass spectrometer and photodiode array (PDA) detector using atmospheric pressure chemical ionisation (APCI) or electrospray ionisation (ESI). High-resolution mass spectra (HRMS) were recorded on a Bruker MicroTOF with ESI or at the EPSRC National Mass Spectrometry service (Swansea, UK) with FAB-MS using *m*-nitrobenzyl alcohol (NBA) as the matrix. HPLC was undertaken using a Waters 717 machine with Autosampler and PDA detector. The column used was a Waters “Symmetry” C18 (packing: 3.5 μm, 4.6 mm×150 mm) with an isocratic mobile phase consisting of H_2_O/CH_3_CN at a flow rate of 1.0 mL min^−1^.

**Method A: Synthesis of adamantyl ethanone ether compounds 6–9**: A suspension of NaH (60 % in mineral oil, 1.2 mmol) in dry THF (4 mL) was treated with the corresponding alcohol (1.1 mmol) at 0 °C. After stirring for 20 min at 0 °C, a solution of adamantan-1-yl bromomethyl ketone (1.0 mmol) in dry THF (4 mL) was added. The mixture was stirred for 2 h at 0 °C then at RT for 12 h. After quenching with water (10 mL), the mixture was extracted with Et_2_O (2×30 mL). The organic phase was washed with brine, dried over MgSO_4_, filtered and concentrated in vacuo. The crude product was purified by flash chromatography (hexane/EtOAc or CH_2_Cl_2_/EtOAc; gradient elution).

**1-(Adamantan-1-yl)-2-(thiophen-2-ylmethoxy)ethanone (6)**: An off-white solid (79 mg, 27 %): mp: 57–58 °C; ^1^H NMR (270 mhz, CDCl_3_): *δ*=1.56–1.79 (m, 12 H), 2.21 (br s, 3 H), 4.30 (s, 2 H), 4.75 (s, 2 H), 6.98 (m, 2 H), 7.30 ppm (m, 1 H); LC/MS (APCI): *m/z* 291 [*M*+H]^+^; HRMS-FAB: *m/z* [*M*+Na]^+^ calcd for C_17_H_22_O_2_SNa: 313.1238, found: 313.1226; HPLC: *t*_R_=2.98 min (99 %) in 10 % H_2_O/CH_3_CN.

**1-(Adamantan-1-yl)-2-(thiophen-3-ylmethoxy)ethanone (7)**: A white solid (68 mg, 23 %): mp: 58–60 °C; ^1^H NMR (270 mhz, CDCl_3_): *δ*=1.63–1.79 (m, 6 H), 1.80 (d, *J=*3.0 Hz, 6 H), 2.01 (br s, 3 H), 4.28 (s, 2 H), 4.58 (s, 2 H), 7.10 (dd, *J=*1.5, 5.0 Hz, 1 H), 7.22 (m, 1 H,), 7.30 ppm (1 H, dd, *J=*3.0, 5.0 Hz); LC/MS (APCI): *m/z* 313 [*M*+Na]^+^; HRMS-FAB: *m/z* [*M*+H]^+^ calcd for C_17_H_23_O_2_S: 291.1413, found: 291.1407; HPLC: *t*_R_=3.26 min (98 %) in 10 % H_2_O/CH_3_CN.

**1-(Adamantan-1-yl)-2-(1,3-thiazol-2-ylmethoxy)ethanone (8)**: A white solid (81 mg, 28 %): mp: 89–91 °C; ^1^H NMR (270 mhz, CDCl_3_): *δ*=1.58–1.74 (m, 6 H), 1.76 (d, *J*=2.8 Hz, 6 H), 1.98 (br s, 3 H), 4.28 (s, 2 H), 4.76 (s, 2 H), 7.75 (s, 1 H), 8.77 ppm (s, 1 H); LC/MS (APCI): *m/z* 292 [*M*+H]^+^; HRMS-FAB: *m/z* [*M*+H]^+^ calcd for C_16_H_22_NO_2_S: 292.1371, found: 292.1372; HPLC: *t*_R_=2.4 min (98 %) in 10 % H_2_O/CH_3_CN.

**1-(Adamantan-1-yl)-2-[(4-methyl-1,3-thiazol-2-yl)methoxy]ethanone (9)**: A white solid (165 mg, 54 %): mp: 77–80 °C; ^1^H NMR (270 mhz, CDCl_3_): *δ*=1.57–1.70 (m, 6 H), 1.75 (d, *J*=2.8 Hz, 6 H), 1.96 (br s, 3 H), 2.36 (s, 3 H), 4.37 (s, 2 H), 4.75 (s, 2 H), 6.82 ppm (s, 1 H); LC/MS (APCI): *m/z* 306 [*M*+H]^+^; HRMS-FAB: *m/z* [*M*+H]^+^ calcd for C_17_H_24_NO_2_S: 306.1528, found 306.1531; HPLC: *t*_R_=2.6 min (97 %) in 10 % H_2_O/CH_3_CN.

**Method B: Synthesis of the adamantyl ethanone sulfanyl derivatives 10–13**: A solution of adamantan-1-yl bromomethyl ketone (1 equiv) in CH_3_CN (15 mL) was treated with the corresponding mercaptan (1.1 equiv), followed by Et_3_N (3 equiv), and the mixture was stirred at RT overnight. 2-Chloro-tritylchloride resin (1.1 equiv, 1.6 mmol g^−1^) was added and the mixture was stirred for 2 h, filtered and concentrated in vacuo to give the crude product, which was purified with flash chromatography (hexane/EtOAc or CH_2_Cl_2_/EtOAc; gradient elution).

**1-(Adamantan-1-yl)-2-(thiophen-2-ylmethylsulfanyl)ethanone (10)**: A semi-solid (160 mg, 52 %): ^1^H NMR (270 mhz, CDCl_3_): *δ*=1.62–1.81 (m, 12 H), 2.02 (br s, 3 H), 3.32 (s, 2 H), 3.95 (s, 2 H), 6.88–6.94 (m, 2 H), 7.20 ppm (dd, *J*=5.0, 1.3 Hz, 1 H); LC/MS (ESI): *m/z* 307 [*M*+H]^+^; HRMS-ESI: *m/z* [*M*+Na]^+^ calcd for C_17_H_22_OS_2_Na: 329.1010, found: 329.0982; HPLC: *t*_R_=5.1 min (97 %) in 20 % H_2_O/CH_3_CN.

**1-(Adamantan-1-yl)-2-(furan-2-ylmethylsulfanyl)-ethanone (11)**: A semi-solid (180 mg, 62 %): ^1^H NMR (270 mhz, CDCl_3_): *δ*=1.69–1.83 (m, 6 H), 1.82 (d, *J*=2.2 Hz, 6 H), 2.03 (br s, 3 H), 3.33 (s, 2 H), 3.75 (s, 2 H), 6.18–6.20 (m, 1 H), 6.29 (dd, *J*=3.1, 1.7 Hz, 1 H), 7.35 ppm (dd, *J*=2.0, 1.0 Hz, 1 H); LC/MS (ESI): *m/z* 291 [*M*+H]^+^; HRMS-ESI: *m/z* [*M*+Na]^+^ calcd for C_17_H_22_O_2_SNa: 313.1238, found: 313.1221; HPLC: *t*_R_=3.6 min (99 %) in 10 % H_2_O/CH_3_CN.

**1-(Adamantan-1-yl)-2-[(1,3-thiazol-2-ylmethyl)sulfanyl]ethanone (12)**: Yellow crystalline solid (170 mg, 55 %): mp: 55–58 °C; ^1^H NMR (270 mhz, CDCl_3_): *δ*=1.60–1.90 (m, 12 H), 2.03 (s, 3 H), 3.32 (s, 2 H), 3.90 (s, 2 H), 7.22 (s, 1 H), 8.77 ppm (s, 1 H); LC/MS (APCI): *m/z* 308 [*M*+H]^+^; HRMS-FAB: *m/z* [*M*+Na]^+^ calcd for C_16_H_21_NNaOS_2_: 330.0962, found: 330.0965; HPLC: *t*_R_=2.6 min (98 %) in 10 % H_2_O/CH_3_CN.

**1-(Adamantan-1-yl)-2-{[(4-methyl-1,3-thiazol-2-yl)methyl]sulfanyl}ethanone (13)**: Yellow oil (178 mg, 55 %): ^1^H NMR (270 mhz, CDCl_3_): *δ*=1.55–1.88 (m, 6 H), 1.81 (d, *J=*2.7 Hz, 6 H), 2.03 (br s, 3 H), 2.40 (s, 3 H), 3.52 (s, 2 H), 3.99 (s, 2 H), 6.80 ppm (s, 1 H); LC/MS (APCI): *m/z* 322 [*M*+H]^+^; HRMS-FAB: *m/z* [*M*+Na]^+^ calcd for C_17_H_23_NNaOS_2_: 344.1119, found: 344.1120; HPLC: *t*_R_=3.7 min (97 %) in 10 % H_2_O/CH_3_CN.

**Method C: Synthesis of the adamantyl ethanone sulfoxide and sulfone derivatives 14–17**: A cold solution of the corresponding sulfanyl derivative (1 equiv) in CH_2_Cl_2_ (10 mL) was treated with *m*-CPBA (2 equiv), and the mixture was stirred at −10 °C to 0 °C for 40 min. The mixture was then partitioned between CH_2_Cl_2_ and 5 % aq Na_2_CO_3_, and the organic phase was washed with brine, dried over MgSO_4_, filtered and concentrated in vacuo to give the crude product. The sulfoxide and sulfone were separated using flash chromatography (EtOAc/CH_2_Cl_2_; gradient elution).

**1-(Adamantan-1-yl)-2-(thiophen-2-ylmethanesulfinyl)ethanone (14)**: A white solid (115 mg, 36 %): mp: 95–98 °C; ^1^H NMR (270 mhz, CDCl_3_): *δ*=1.62–1.81 (m, 12 H), 2.05 (br s, 3 H), 3.57 (d, *J*=16 Hz, 1 H), 3.86 (d, *J*=16 Hz, 1 H), 4.36 (AB, 2 H), 7.02–7.07 (m, 2 H), 7.33 ppm (dd, *J*=5.2, 1.5 Hz, 1 H); LC/MS (APCI): *m/z* 321 [*M*−H]^+^; HRMS-FAB: *m/z* [*M*+Na]^+^ calcd for C_17_H_22_O_2_S_2_Na: 345.0959, found: 345.0938; HPLC: *t*_R_=2.66 min (>99 %) in 20 % H_2_O/CH_3_CN. **1-(Adamantan-1-yl)-2-(thiophen-2-ylmethanesuphonyl)ethanone (16)**: A white solid (68 mg, 21 %): mp: 96–98 °C; ^1^H NMR (270 mhz, CDCl_3_): *δ*=1.63–1.72 (m, 6 H), 1.78 (d, *J*=2.7 Hz, 6 H), 2.07 (br s, 3 H), 3.95 (s, 2 H), 4.73 (s, 2 H), 7.03 (dd, *J*=5.2, 3.4 Hz, 1 H), 7.20 (dd, *J*=3.4, 1.3 Hz, 1 H), 7.36 ppm (dd, *J*=5.2, 1.3 Hz, 1 H); LC/MS (APCI): *m/z* 337 [*M*^+^−H]; HRMS-FAB: *m/z* [*M*+Na]^+^ calcd for C_17_H_22_NaO_3_S_2_: 361.0908, found: 361.0889; HPLC: *t*_R_=2.4 min (>99 %) in 10 % H_2_O/CH_3_CN.

**1-(Adamantan-1-yl)-2-(furan-2-ylmethanesulfinyl)ethanone (15)**: A white solid (100 mg, 33 %): mp: 75–79 °C; ^1^H NMR (270 mhz, CDCl_3_): *δ*=1.56–1.84 (m, 12 H), 2.06 (br s, 3 H), 3.67 (d, *J*=15 Hz, 1 H), 3.93 (d, *J*=15 Hz, 1 H), 4.20 (AB, 2 H), 6.04–6.44 (m, 2 H), 7.44 ppm (dd, *J*=1.8, 0.7 Hz, 1 H); LC/MS (APCI): *m/z* 307 [*M*+H]^+^; HRMS-FAB: *m/z* [*M*+Na]^+^ calcd for C_17_H_22_O_3_SNa: 329.1187, found: 329.1164; HPLC: *t*_R_=2.54 min (>99 %) in 20 % H_2_O/CH_3_CN. **1-(Adamantan-1-yl)-2-[(furan-2-ylmethane)sulfonyl]ethanone (17)**: An off-white solid (166 mg, 52 %): mp: 99–100 °C; ^1^H NMR (270 mhz, CDCl_3_): *δ*=1.65–1.85 (m, 12 H), 2.07 (br s, 3 H), 4.02 (s, 2 H), 4.61 (s, 2 H), 6.40 (t, *J*=2.5 Hz, 1 H), 6.51 (d, *J*=3.0 Hz, 1 H), 7.45 ppm (d, *J*=2.7 Hz, 1 H); LC/MS (APCI): *m/z* 323 [*M*+H]^+^; HRMS-FAB: *m/z* [*M*+H]^+^ calcd for C_17_H_23_O_4_S: 323.1317, found: 323.1326; HPLC: *t*_R_=2.0 min (>99 %) in 10 % H_2_O/CH_3_CN.

**Methyl 5-({[2-(adamantan-1-yl)-2-oxoethyl]sulfanyl}methyl)furan-2-carboxylate (18)**: A solution of 2-(acetylsulfanyl)-1-(adamantan-1-yl)ethanone (1.76 g, 6.97 mmol) in acetone (20 mL) was treated with 1 n NaOH (7 mL). The mixture was stirred at RT under nitrogen for 1 h. Methyl 5-(chloromethyl)furan-2-carboxylate (1.2 g, 6.87 mmol) in CH_3_CN/Et_3_N (20–8 mL) was added. After stirring at RT for 12 h, the mixture was partitioned between EtOAc and brine. The organic phase was washed with brine, dried over MgSO_4_, filtered and concentrated in vacuo. Purification with flash column (EtOAc/petrolium ether; gradient elution) yielded a colourless oil (1.24 g, 52 %): ^1^H NMR (270 mhz, CDCl_3_): *δ*=1.65–1.80 (m, 6 H), 1.82 (d, *J*=2.7 Hz, 6 H), 2.02 (br s, 3 H), 3.36 (s, 2 H), 3.75 (s, 2 H), 3.86 (s, 3 H), 6.34 (d, *J*=3.5 Hz, 1 H), 7.05 ppm (d, *J*=3.5 Hz, 1 H); LC/MS (ESI): *m/z* 371 [*M*+Na]^+^; HRMS-FAB: *m/z* [*M*+Na]^+^ calcd for C_19_H_24_NaO_4_S: 371.1293, found: 371.1255; HPLC: *t*_R_=2.86 min (98 %) in 10 % H_2_O/CH_3_CN.

**5-(((2-(Adamantan-1-yl)-2-oxoethyl)thio)methyl)furan-2-carboxylic acid (19)**: A solution of **18** (860 mg, 2.47 mmol) in MeOH/H_2_O (10–3 mL) was treated with KOH (345 mg) at RT and stirred for 12 h. Compound **19** was isolated by neutralisation with 2 n HCl and filtration as a white solid (620 mg, 75 %): mp: 126–127 °C; ^1^H NMR (270 mhz, CDCl_3_): *δ*=1.62–1.80 (m, 6 H), 1.83 (d, *J*=2.7 Hz, 6 H), 2.03 (br s, 3 H), 3.38 (s, 2 H), 3.78 (s, 2 H), 6.39 (d, *J=*3.3 Hz, 1 H), 7.23 ppm (d, *J=*3.3 Hz, 1 H); LC/MS (ESI): *m/z* 357 [*M*+Na]^+^; HRMS-ESI: *m/z* [*M*+Na]^+^ calcd for C_18_H_22_NaO_4_S: 357.1136, found: 357.1122; HPLC: *t*_R_=1.80 min (97 %) in 10 % H_2_O/CH_3_CN.

**5-({[2-(Adamantan-1-yl)-2-oxoethyl]sulfanyl}methyl)-*N,N*-dimethylfuran-2-carboxamide (20)**: A solution of **19** (293 mg, 0.88 mmol) in CH_2_Cl_2_ (10 mL) was treated with EDCI (184 mg, 0.96 mmol), HOBt (65 mg, 0.48 mmol) and DMAP (20 mg), followed by Et_3_N (0.28 mL, 2.0 mmol). The mixture was stirred at RT for 10 min and then treated with *N*,*N*-dimethylamine (DMA)/tetrahydrofuran (THF) (2 m, 1 mL, 2.0 mmol). After stirring at RT for 16 h, the mixture was partitioned between CH_2_Cl_2_ and 2 % HCl solution. The organic phase was washed with 5 % aq NaHCO_3_ and brine, dried over MgSO_4_, filtered and concentrated in vacuo. Purification by flash column (CH_2_Cl_2_/EtOAc; gradient elution) yielded **20** as colourless oil (185 mg, 58 %): ^1^H NMR (270 mhz, CDCl_3_): *δ*=1.62–1.78 (m, 6 H), 1.81 (d, *J*=2.7 Hz, 6 H), 2.02 (s, 3 H), 3.09 (br s, 3 H), 3.24 (br s, 3 H), 3.33 (s, 2 H), 3.75 (s, 2H_2_), 6.28 (d, *J*=3.3 Hz, 1 H), 6.91 ppm (d, *J*=3.3 Hz, 1 H); LC/MS (ESI): *m*/*z* 384 [*M*+Na]^+^; HRMS-ESI: *m/z* [*M*+H]^+^ calcd for C_20_H_28_NO_3_S: 362.1790, found: 362.1770; HPLC: *t*_R_=2.43 min (97 %) in 10 % H_2_O/CH_3_CN.

**Methyl 5-({[2-(adamantan-1-yl)-2-oxoethane]sulfinyl}methyl)furan-2-carboxylate (21)**: Prepared using method C. A white solid (35 mg, 21 %): mp: 119–120 °C; ^1^H NMR (270 mhz, CDCl_3_): *δ*=1.64–1.78 (m, 6 H), 1.80 (d, *J*=2.7 Hz, 6 H), 2.06 (br s, 3 H), 3.79 (d, *J*=16 Hz, 1 H), 3.86 (s, 3 H), 3.95 (d, *J*=16 Hz, 1 H), 4.19 (d, *J*=14 Hz, 1 H), 4.30 (d, *J*=14 Hz, 1 H), 6.57 (d, *J*=3.5 Hz, 1 H), 7.18 ppm (d, *J*=3.6 Hz, 1 H); LC/MS (ESI): *m/z* 387 [*M*+Na]^+^; HRMS-ESI: *m/z* [*M*+H]^+^ calcd for C_19_H_25_O_5_S: 365.1423, found: 365.1410; HPLC: *t*_R_=1.92 min (>99 %) in 10 % H_2_O/CH_3_CN. **Methyl 5-({[2-(adamantan-1-yl)-2-oxoethane]sulfonyl}methyl)furan-2-carboxylate (22)**: Prepared using method C. A white solid (101 mg, 60 %): mp: 135–136 °C; ^1^H NMR (270 mhz, CDCl_3_): *δ*=1.64–1.78 (m, 6 H), 2.02 (d, *J*=2.6 Hz, 6 H), 2.07 (br s, 3 H), 3.86 (s, 3 H), 4.10 (s, 2 H), 4.68 (s, 2 H), 6.63 (d, *J*=3.5 Hz, 1 H), 7.16 ppm (d, *J*=3.3 Hz, 1 H); LC/MS (ESI): *m/z* 403 [*M*+Na]^+^; HRMS-ESI: *m/z* [*M*+Na]^+^ calcd for C_19_H_25_O_6_S: 381.1372, found: 381.1360 [*M*+H]^+^; HPLC: *t*_R_=2.01 min (>99 %) in 10 % H_2_O/CH_3_CN.

**5-({[2-(Adamantan-1-yl)-2-oxoethane]sulfonyl}methyl)furan-2-carboxylic acid (23)**: Prepared using method C. A white solid (97 mg, 46 %): mp: 163–165 °C; ^1^H NMR (270 mhz, CDCl_3_): *δ*=1.60–1.79 (m, 12 H), 2.04 (br s, 3 H), 4.15 (s, 2 H), 4.69 (s, 2 H), 6.58 (br s, 1 H), 7.19 (br s, 1 H); LC/MS (ESI): *m/z* 367 [*M*+H]^+^; HRMS-ESI: *m/z* [*M*+H]^+^ calcd for C_18_H_23_O_6_S: 367.1215, found: 367.1209; HPLC: *t*_R_=1.76 min (98 %) in 10 % H_2_O/CH_3_CN.

**5-({[2-(Adamantan-1-yl)-2-oxoethane]sulfinyl}methyl)-*N,N*-dimethylfuran-2-carboxamide (24)**: Prepared using method C. A colourless oil (55 mg, 50 %): ^1^H NMR (270 mhz, CDCl_3_): *δ*=1.62–1.80 (m, 12 H), 2.05 (br, 3 H), 3.07 (br s, 3 H), 3.23 (br s, 3 H), 3.72 (d, *J*=16 Hz, 1 H), 3.97 (s, 3 H), 3.95 (d, *J*=16 Hz, 1 H), 4.19 (d, *J*=15 Hz, 1 H), 4.31 (d, *J*=15 Hz, 1 H), 6.59 (d, *J*=3.5 Hz, 1 H), 6.96 ppm (d, *J*=3.6 Hz, 1 H); LC/MS (ESI): *m/z* 378 [*M*+H]^+^; HRMS-ESI: *m/z* [*M*+H]^+^ calcd for C_20_H_29_NO_4_S: 378.1739, found: 378.1724; HPLC: *t*_R_=1.73 min (>99 %) in 10 % H_2_O/CH_3_CN. **5-({[2-(Adamantan-1-yl)-2-oxoethane]sulfonyl}methyl)-*N,N*-dimethylfuran-2-carboxamide (25)**: Prepared using method C. A white solid (35 mg, 31 %): mp: 134–135 °C; ^1^H NMR (270 mhz, CDCl_3_): *δ*=1.62–1.77 (m, 6 H), 1.79 (d, *J*=2.8 Hz, 6 H), 2.07 (br s, 3 H), 3.06 (s, 3 H), 3.25 (s, 3 H), 4.03 (s, 2 H), 4.65 (s, 2 H), 6.59 (d, *J*=3.3 Hz, 1 H), 7.00 ppm (d, *J*=3.3 Hz, 1 H); LC/MS (ESI): *m/z* 394 [*M*+H]^+^; HRMS-ESI: *m/z* [*M*+H]^+^ calcd for C_20_H_28_NO_5_S: 394.1688, found: 394.1670; HPLC: *t*_R_=1.70 min (98 %) in 10 % H_2_O/CH_3_CN.

**1-(Adamantan-1-yl)-2-(thiophen-2-ylsulfanyl)-ethanone (26)**: Prepared using method B. An Off-white solid (250 mg, 86 %): mp: 69–72 °C; ^1^H NMR (270 mhz, CDCl_3_): *δ*=1.67–1.78 (m, 12 H), 2.02 (br s 3 H), 3.84 (s, 2 H), 6.94 (dd, *J*=5.1, 3.2 Hz, 1 H), 7.15 (dd, *J*=3.3, 1.5 Hz, 1 H), 7.34 ppm (dd, *J*=5.2, 1.5 Hz, 1 H); LC/MS (APCI): *m/z* 293 [*M*+H]^+^; HRMS-FAB: *m/z* [*M*+Na]^+^ calcd for C_16_H_20_OS_2_Na: 315.0853, found: 315.0838; HPLC: *t*_R_=3.6 min (>99 %) in 10 % H_2_O/CH_3_CN.

**1-(Adamantan-1-yl)-2-(1-methyl-1*H*-imidazol-2-ylsulfanyl)-ethanone (27)**: Prepared using method B. An off-white solid (230 mg, 79 %): mp: 65–69 °C; ^1^H NMR (270 mhz, CDCl_3_): *δ*=1.63–1.81 (m, 6 H), 1.82 (d, *J*=2.3 Hz, 6 H), 2.03 (br s, 3 H), 3.64 (s, 3 H), 4,23 (s, 2 H), 6.89 (d, *J*=1.2 Hz, 1 H), 7.01 ppm (d, *J*=1.2 Hz, 1 H); LC/MS (APCI): *m/z* 291 [*M*+H]^+^; HRMS-FAB: *m/z* [*M*+H]^+^ calcd for C_16_H_23_N_2_OS: 291.1531, found: 291.1524; HPLC: *t*_R_=3.0 min (>99 %) in 10 % H_2_O/CH_3_CN.

**1-(Adamantan-1-yl)-2-(4-methyl-4*H*-[1,2,4]triazol-3-ylsulfanyl)-ethanone (28)**: Prepared using method B. A white solid (340 mg, 58 %): mp: 135–136 °C; ^1^H NMR (270 mhz, CDCl_3_): *δ*=1.60–1.80 (m, 6 H), 1.87 (d, *J*=2.8 Hz, 6 H), 2.05 (br s, 3 H), 3.63 (s, 3 H), 4.47 (s, 2 H), 8.09 ppm (s, 1 H); LC/MS (ESI): *m/z* 292 [*M*+H]^+^; HRMS-ESI: *m/z* [*M*+H]^+^ calcd for C_15_H_22_N_3_OS: 292.1484, found: 292.1484; HPLC: *t*_R_=2.0 min (>99 %) in 10 % H_2_O/CH_3_CN.

**1-(Adamantan-1-yl)-2-(5-methyl-[1,3,4]thiadiazol-2-ylsulfanyl)-ethanone (29)**: Prepared using method B. A white solid (670 mg, 87 %): mp: 102–105 °C; ^1^H NMR (270 mhz, CDCl_3_): *δ*=1.65–1.80 (m, 6 H), 1.90 (d, *J*=2.8 Hz, 6 H), 2.06 (br s, 3 H), 2.69 (s, 3 H), 4.48 ppm (s, 2 H); LC/MS (ESI): *m/z* 309 [*M*+H]^+^; HRMS-ESI: *m/z* [*M*+Na]^+^ calcd for C_15_H_20_N_2_OS_2_Na: 331.0915, found: 331.0873; HPLC: *t*_R_=2.7 min (>99 %) in 10 % H_2_O/CH_3_CN.

**5-({[2-(Adamantan-1-yl)-2-oxoethane]sulfinyl}methyl)-*N,N*-dimethylfuran-2-carboxamide (30)**: Prepared using method C. A yellow solid (160 mg, 52 %): mp: 114–116 °C; ^1^H NMR (270 mhz, CDCl_3_): *δ*=1.56–1.75 (m, 12 H), 2.03 (br s, 3 H), 4.06 (d, *J*=15 Hz, 1 H), 4.39 (d, *J*=15 Hz, 1 H), 7.10 (dd, *J*=5.1, 3.6 Hz, 1 H), 7.48 (dd, *J*=3.4, 1.2 Hz, 1 H), 7.65 ppm (dd, *J*=5.0, 1.3 Hz, 1 H); LC/MS (APCI): *m/z* 307 [*M*−H]^+^; HRMS-FAB: *m/z* [*M*+Na]^+^ calcd for C_16_H_20_O_2_S_2_Na: 331.0802, found: 331.0780; HPLC: *t*_R_=2.7 min (>99 %) in 10 % H_2_O/CH_3_CN. **5-({[2-(Adamantan-1-yl)-2-oxoethane]sulfonyl}methyl)*-N,N*-dimethylfuran-2-carboxamide (34)**: Prepared using method C. A white solid (90 mg, 28 %): mp: 117.5–119 °C; ^1^H NMR (270 mhz, CDCl_3_): *δ*=1.61–1.74 (m, 6 H), 1.75 (d, *J*=2.7 Hz, 6 H), 2.04 (br s, 3 H), 4.36 (s, 2 H), 7.14 (dd, *J*=5.0, 3.7 Hz, 1 H), 7.72–7.76 ppm (m, 2 H); LC/MS (APCI): *m/z* 323 [*M*−H]^+^; HRMS-FAB: *m/z* [*M*+Na]^+^ calcd for C_16_H_20_O_3_S_2_Na: 347.0752, found: 347.0721; HPLC: *t*_R_=2.25 min (98 %) in 10 % H_2_O/CH_3_CN.

**1-(Adamantan-1-yl)-2-(1-methyl-1*H*-imidazole-2-sulfinyl)ethanone (31)**: Prepared using method C. A yellow solid (170 mg, 56 %): mp: 86–89 °C; ^1^H NMR (270 mhz, CDCl_3_): *δ*=1.63–1.85 (m, 12 H), 2.04 (br s, 3 H), 3.93 (s, 3 H), 4.51 (d, *J*=17 Hz, 1 H), 4.91 (d, *J*=17 Hz, 1 H), 6.98 (d, *J*=1.0 Hz, 1 H), 7.15 ppm (d, *J*=1.0 Hz, 1 H); LC/MS (APCI): *m/z* 307 [*M*+H]^+^; HRMS-FAB: *m/z* [*M*+H]^+^ calcd for C_16_H_23_N_2_O_2_S: 307.1480, found: 307.1477; HPLC: *t*_R_=2.6 min (97 %) in 10 % H_2_O/CH_3_CN. **1-(Adamantan-1-yl)-2-(1-methyl-1*H*-imidazole-2-sulfonyl)ethanone (35)**: Prepared using method C. A white solid (66 mg, 20 %): mp: 124–125 °C; ^1^H NMR (270 mhz, CDCl_3_): *δ*=1.61–1.74 (m, 12 H), 2.04 (br s, 3 H), 4.01 (s, 3 H), 4.58 (s, 2 H), 7.14 ppm (d, *J*=1.0 Hz, 1 H); LC/MS (APCI): *m/z* 321 [*M*−H]^+^; HRMS-FAB: *m/z* [*M*+H]^+^ calcd for C_16_H_23_N_2_O_3_S: 323.1429, found: 323.1418; HPLC: *t*_R_=2.0 min (99 %) in 10 % H_2_O/CH_3_CN.

**1-(Adamantan-1-yl)-2-(4-methyl-4*H*-[1,2,4]triazole-3-sulfinyl)ethanone (32)**: Prepared using method C. A white solid (180 mg, 59 %): mp: 124.5–127 °C; ^1^H NMR (270 mhz, CDCl_3_): *δ*=1.62–1.84 (m, 12 H), 2.07 (br, 3 H), 3.97 (s, 3 H), 4.62 (d, *J*=15 Hz, 1 H), 5.09 (d, *J*=15 Hz, 1 H), 8.20 ppm (s, 1 H); LC/MS (ESI): *m/z* 306 [*M*−H]^+^; HRMS-ESI: *m/z* [*M*+Na]^+^ calcd for C_15_H_21_N_3_O_2_SNa: 330.1252, found: 330.1223; HPLC: *t*_R_=2.1 min (97 %) in 10 % H_2_O/CH_3_CN. **1-(Adamantan-1-yl)-2-(4-methyl-4*H*-[1,2,4]triazole-3-sulfonyl)ethanone (36)**: Prepared using method C. A white solid (85 mg, 26 %): mp: 149–150 °C; ^1^H NMR (270 mhz, CDCl_3_): *δ*=1.60–1.80 (m, 12 H), 2.05 (br s, 3 H), 4.02 (s, 3 H), 4.71 (s, 2 H), 8.17 ppm (s, 1 H); LC/MS (ESI): *m/z* 322 [*M*−H]^+^; HRMS-ESI: *m/z* [*M*+Na]^+^ calcd for C_15_H_21_N_3_O_3_SNa: 346.1201, found: 346.1160; HPLC: *t*_R_=1.7 min (97 %) in 10 % H_2_O/CH_3_CN.

**1-(Adamantan-1-yl)-2-(5-methyl-[1,3,4]thiadiazole-2-sulfinyl)ethanone (33)**: Prepared using method C. A white solid (180 mg, 56 %): mp: 127–129 °C; ^1^H NMR (270 mhz, CDCl_3_): *δ*=1.66–1.79 (m, 6 H), 1.82 (d, *J*=2.7 Hz, 6 H), 2.06 (br s, 3 H), 2.85 (s, 3 H), 4.44 ppm (q, *J*=15 Hz, 2 H); LC/MS (ESI): *m/z* 325 [*M*+H]^+^; HRMS-ESI: *m/z* [*M*+Na]^+^ calcd for C_15_H_20_N_2_O_2_S_2_Na: 347.0864, found: 347.0817; HPLC: *t*_R_=2.0 min (99 %) in 10 % H_2_O/CH_3_CN. **1-(Adamantan-1-yl)-2-(5-methyl-[1,3,4]thiadiazole-2-sulfonyl)ethanone (37)**: Prepared using method C. A white solid (60 mg, 18 %): mp: 111–113 °C; ^1^H NMR (270 mhz, CDCl_3_): *δ*=1.65–1.75 (m, 6 H), 1.76 (d, *J*=2.7 Hz, 6 H), 2.06 (br s, 3 H), 2.89 (s, 3 H), 4.75 ppm (s, 2 H); LC/MS (ESI): *m/z* 341 [*M*+H]^+^; HRMS-ESI: *m/z* [*M*+Na]^+^ calcd for C_15_H_20_N_2_O_3_S_2_Na: 363.0813, found: 363.0769; HPLC: *t*_R_=2.0 min (97 %) in 10 % H_2_O/CH_3_CN.

**1-(Adamantan-1-yl)-2-[(4,5-dimethyl-1,2,4-triazol-3-yl)sulfanyl]ethanone (38)**: A mixture of 2-acetyl-*N*-methylhydrazinecarbothioamide (441 mg, 3 mmol) in 2 n aq NaOH (4 mL) was refluxed under nitrogen for 4 h, cooled to RT and concentrated in vacuo. The residue was redissolved in CH_3_CN (12 mL), treated with adamantan-1-yl bromomethyl ketone (514 mg, 2 mmol) and stirred at RT overnight. The reaction was partitioned between CH_2_Cl_2_ and water, and the organic phase was washed with brine, dried over MgSO_4_, filtered and concentrated in vacuo. Purification by flash column (CH_2_Cl_2_/MeOH; gradient elution) yielded **38** as white solid (290 mg, 41 %): mp: 113–114 °C, ^1^H NMR (270 mhz, CDCl_3_): *δ*=1.62–1.80 (m, 6 H), 1.85 (d, *J*=2.8 Hz, 6 H), 2.15 (br s, 3 H), 2.39 (s, 3 H), 3.49 (s, 3 H), 4.39 ppm (s, 2 H); LC/MS (ESI): *m/z* 306 [*M*+H]^+^; HRMS-ESI: *m/z* [*M*+H]^+^ calcd for C_16_H_24_N_3_OS: 306.1640, found: 306.1627; HPLC: *t*_R_=1.8 min (99 %) in 10 % H_2_O/CH_3_CN.

**1-(Adamantan-1-yl)-2-{[5-methyl-4-(propan-2-yl)-4*H*-1,2,4-triazol-3-yl]sulfanyl}ethanone (39)**: A mixture of 2-acetyl-*N*-isopropylhydrazinecarbothioamide (780 mg, 4.46 mmol) in 2 n aq NaOH (5 mL) was refluxed under nitrogen for 6 h, cooled to RT and concentrated in vacuo. The residue was redissolved in CH_3_CN (15 mL), treated with adamantan-1-yl bromomethyl ketone (900 mg, 3.5 mmol) and stirred at RT overnight. The reaction was partitioned between CH_2_Cl_2_ and water, and the organic phase was washed with brine, dried over MgSO_4_, filtered and concentrated in vacuo. Purification by flash column (CH_2_Cl_2_/EtOAc; gradient elution) yielded **39** as white solid (510 mg, 44 %): mp: 119–121 °C, ^1^H NMR (270 mhz, CDCl_3_): *δ*=1.49 (d, *J*=6.8 Hz, 6 H), 1.65–1.85 (m, 6 H), 1.87 (d, *J*=2.5 Hz, 6 H), 2.03 (br s, 3 H), 2.45 (s, 3 H), 4.45 ppm (m, 3 H); LC/MS (ESI): *m/z* 334 [*M*+H]^+^; HRMS-ESI: *m/z* [*M*+H]^+^ calcd for C_18_H_28_N_3_OS: 334.1953, found: 334.1953; HPLC: *t*_R_=2.1 min (99 %) in 10 % H_2_O/CH_3_CN.

**1-(Adamantan-1-yl)-2-[(5-cyclopropyl-4-methyl-4*H*-1,2,4-triazol-3-yl)sulfanyl]ethanone (40)**: A mixture of 2-(cyclopropanecarbonyl)-*N*-methylhydrazinecarbothioamide (700 mg, 4.05 mmol) in 2 n aq NaOH (5 mL) was refluxed under nitrogen for 5 h, cooled to RT and concentrated in vacuo. The residue was redissolved in CH_3_CN (5 mL), treated with adamantan-1-yl bromomethyl ketone (771 mg, 3.0 mmol) and stirred at RT overnight. The reaction was partitioned between CH_2_Cl_2_ and water, and the organic phase was washed with brine, dried over MgSO_4_, filtered and concentrated in vacuo. Purification by flash column (CH_2_Cl_2_/EtOAc; gradient elution) yielded **40** as white solid (390 mg, 39 %): mp: 96–97.5 °C, ^1^H NMR (270 mhz, CDCl_3_): *δ*=1.00 (m, 4 H), 1.60–1.80 (m, 7 H), 1.83 (d, *J*=2.8 Hz, 6 H), 2.01 (br s, 3 H), 3.57 (s, 3 H), 4.35 ppm (s, 2 H); LC/MS (ESI): *m/z* 332 [*M*+H]^+^; HRMS-ESI: *m/z* [*M*+H]^+^ calcd for C_18_H_26_N_3_O_S_: 332.1796, found: 332.1796; HPLC: *t*_R_=1.9 min (99 %) in 10 % H_2_O/CH_3_CN.

**1-(Adamantan-1-yl)-2-{[5-(methoxymethyl)-4-methyl-4*H*-1,2,4-triazol-3-yl]sulfanyl}ethanone (41)**: A solution of 4-methyl-3-thiosemicarbazide (526 mg, 5.0 mmol) in CH_2_Cl_2_ (10 mL) was treated with pyridine (1.2 mL), followed by metholoxyacetyl chloride (0.46 mL, 5 mmol) at 0 °C. The mixture was stirred at RT overnight, then concentrated in vacuo. The residue was redissolved in 2 n aq NaOH (6 mL), and the solution was refluxed under nitrogen for 6 h. The reaction was cooled to RT, treated with adamantan-1-yl bromomethyl ketone (643 mg, 2.5 mmol), and stirred at RT overnight. The mixture was partitioned between CH_2_Cl_2_ and brine, and the organic phase was washed with brine, dried over MgSO_4_, filtered and concentrated in vacuo. Purification by flash column (CH_2_Cl_2_/CH_3_OH; gradient elution) yielded **41** as white solid (550 mg, 66 %): mp: 109–111 °C; ^1^H NMR (270 mhz, CDCl_3_): *δ*=1.60–1.82 (m, 6 H), 1.86 (d, *J*=2.8 Hz, 6 H), 2.04 (br s, 3 H), 3.34 (s, 3 H), 3.59 (s, 3 H), 4.44 (s, 2 H), 4.58 ppm (s, 2 H); LC/MS (ESI): *m/z* 336 [*M*+H]^+^; HRMS-ESI: *m/z* [*M*+H]^+^ calcd for C_17_H_26_N_3_O_2_S: 336.1745, found: 336.1730; HPLC: *t*_R_=1.8 min (99 %) in 10 % H_2_O/CH_3_CN.

**Methyl 2-(5-{[2-(adamantan-1-yl)-2-oxoethyl]sulfanyl}-4-methyl-4*H*-1,2,4-triazol-3-yl)acetate (42)**: A solution of 4-methyl-3-thiosemicarbazide (316 mg, 3.0 mmol) in CH_2_Cl_2_ (10 mL) was treated with pyridine (1.2 mL), followed by 2-cyanoacetyl chloride (312 mg, 3 mmol) at 0 °C. The mixture was stirred at RT overnight, and then concentrated in vacuo. The residue was redissolved in 2 n aq NaOH (6 mL) and refluxed under nitrogen for 6 h. The reaction was cooled to RT, treated with adamantan-1-yl bromomethyl ketone (643 mg, 2.5 mmol), and stirred at RT overnight. The mixture was partitioned between CH_2_Cl_2_ and brine, and the organic phase was washed with brine, dried over MgSO_4_, filtered and concentrated in vacuo. The crude residue was redissolved in MeOH (10 mL) and treated with HCl (37 %, 0.5 mL) and the mixture was refluxed for 6 h. Aqueous work-up gave a clear oil, and subsequent purification by flash column (CH_2_Cl_2_/CH_3_OH; gradient elution) yielded **42** as white solid (344 mg, 38 %): mp: 135–137 °C; ^1^H NMR (270 mhz, CDCl_3_): *δ*=1.59–1.81 (m, 6 H), 1.87 (d, *J*=2.8 Hz, 6 H), 2.02 (br s, 3 H), 3.52 (s, 3 H), 3.72 (s, 3 H), 3.86 (s, 2 H), 4.46 ppm (s, 2 H); LC/MS (ESI): *m/z* 364[*M*+H]^+^; HRMS-ESI: *m/z* [*M*+H]^+^ calcd for C_18_H_26_N_3_O_3_S: 364.1695, found: 364.1698; HPLC: *t*_R_=1.1 min (99 %) in 10 % H_2_O/CH_3_CN.

**1-(Adamantan-1-yl)-2-[(4,5-dicyclopropyl-4*H*-1,2,4-triazol-3-yl)sulfanyl]ethanone (43)**: Prepared using method B. A white solid (295 mg, 65 %): mp: 117–119 °C; ^1^H NMR (270 mhz, CDCl_3_): *δ*=1.02 (m, 8 H), 1.59–1.83 (m, 7 H), 1.82 (d, *J*=2.8 Hz, 6 H), 2.01 (br s, 3 H), 3.15 (m, 1 H), 4.39 ppm (s, 2 H); LC/MS (ESI): *m/z* 358 [*M*+H]^+^; HRMS-ESI: *m/z* [*M*+H]^+^ calcd for C_20_H_28_N_3_OS: 358.1953, found: 358.1943; HPLC: *t*_R_=1.7 min (98 %) in 10 % H_2_O/CH_3_CN.

**1-(Adamantan-1-yl)-2-{[4-methyl-5-(thiophen-2-yl)-4*H*-1,2,4-triazol-3-yl]sulfanyl}ethanone (44)**: Prepared using method B. A white solid (395 mg, 53 %): mp: 156–157 °C; ^1^H NMR (270 mhz, CDCl_3_): *δ*=1.66–1.82 (m, 6 H), 1.88 (d, *J*=2.8 Hz, 6 H), 2.05 (br s, 3 H), 3.73 (s, 3 H), 4.46 (s, 2 H), 7.14 (t, *J*=4.9 Hz, 1 H), 7.42 (d, *J*=4.8 Hz, 1 H), 7.47 ppm (d, *J*=4.9 Hz,1 H); LC/MS (ESI): *m/z* 374 [*M*+H]^+^; HRMS-ESI: *m/z* [*M*+H]^+^ calcd for C_19_H_24_N_3_OS_2_: 374.1361, found: 374.1362; HPLC: *t*_R_=2.1 min (>99 %) in 10 % H_2_O/CH_3_CN.

**1-(Adamantan-1-yl)-2-{[5-(dimethylamino)-1,3,4-thiadiazol-2-yl]sulfanyl}ethanone (45)**: A solution of *N,N*-dimethylhydrazinecarbothioamide (477 mg, 4 mmol) in DMF (6 mL) was treated with Et_3_N (1 mL), followed by CS_2_ (0.4 mL). The mixture was stirred at RT overnight and then at 60 °C for 5 h. The reaction was cooled to RT, treated with adamantan-1-yl bromomethyl ketone (514 mg, 2.0 mmol) and stirred at RT overnight. The mixture was partitioned between EtOAc and brine, and the organic phase was washed with brine, dried over MgSO_4_, filtered and concentrated in vacuo. Purification by flash column (CH_2_Cl_2_/EtOAc; gradient elution) yielded **45** as an off-white solid (365 mg, 54 %): mp: 150–151 °C; ^1^H NMR (270 mhz, CDCl_3_): *δ*=1.65–1.82 (m, 6 H), 1.85 (d, *J*=2.8 Hz, 6 H), 2.03 (br s, 3 H), 3.11 (s, 6 H), 4.40 ppm (s, 2 H); LC/MS (ESI): *m/z* 338 [*M*+H]^+^; HRMS-ESI: *m/z* [*M*+H]^+^ calcd for C_16_H_24_N_3_OS_2_: 338.1361, found: 338.1353; HPLC: *t*_R_=2.4 min (99 %) in 10 % H_2_O/CH_3_CN.

**1-(Adamantan-1-yl)-2-{[5-(methylsulfanyl)-1,3,4-thiadiazol-2-yl]sulfanyl}ethanone (46)**: Prepared using method B. A white solid (310 mg, 91 %): mp: 133.5–135 °C; ^1^H NMR (270 mhz, CDCl_3_): *δ*=1.65–1.83 (m, 6 H), 1.89 (d, *J*=2.7 Hz, 6 H), 2.06 (br s, 3 H), 2.72 (s, 3 H), 4.47 ppm (s, 2 H); LC/MS (ESI): *m/z* 341 [*M*+H]^+^; HRMS-ESI: *m/z* [*M*+H]^+^ calcd for C_15_H_21_N_2_OS_3_: 341.0816, found: 341.0807; HPLC: *t*_R_=3.1 min (98 %) in 10 % H_2_O/CH_3_CN.

**1-(Adamantan-1-yl)-2-{[5-(ethylsulfanyl)-1,3,4-thiadiazol-2-yl]sulfanyl}ethanone (47)**: Prepared using method B. A colourless oil (277 mg, 71 %): ^1^H NMR (270 mhz, CDCl_3_): *δ*=1.30 (t, *J*=7.2 Hz, 3 H,),1.63–1.81 (m, 6 H), 1.88 (d, *J*=2.7 Hz, 6 H), 2.03 (br s, 3 H), 2.73 (q, *J*=7.2 Hz, 2 H), 4.47 ppm (s, 2 H); LC/MS (ESI): *m/z* 355 [*M*+H]^+^; HRMS-ESI: *m/z* [*M*+H]^+^ calcd for C_16_H_23_N_2_OS_3_: 355.0973, found: 355.0968; HPLC: *t*_R_=2.7 min (98 %) in 10 % H_2_O/CH_3_CN.

**1-(Adamantan-1-yl)-2-[(5-amino-1,3,4-thiadiazol-2-yl)sulfanyl]ethanone (48)**: Prepared using method B. A white solid (504 mg, 91 %): mp: 198–199 °C; ^1^H NMR (270 mhz, CDCl_3_): *δ*=1.62–1.80 (m, 6 H), 1.85 (d, *J*=2.8 Hz, 6 H), 2.15 (br s, 3 H), 2.39 (s, 3 H), 3.49 (s, 3 H), 4.39 ppm (s, 2 H); LC/MS (ESI): *m/z* 310 [*M*+H]^+^; HRMS-ESI: *m/z* [*M*+H]^+^ calcd for C_14_H_20_N_3_OS_2_: 310.1048, found: 310.1037; HPLC: *t*_R_=2.0 min (99 %) in 10 % H_2_O/CH_3_CN.

**1-(Adamantan-1-yl)-2-(5-amino-1,3,4-thiadiazole-2-sulfonyl)ethanone (49)**: Prepared using method C. A white solid (165 mg, 75 %): mp: 202–204 °C; ^1^H NMR (270 mhz, CDCl_3_): *δ*=1.62–1.80 (m, 6 H), 1.85 (d, *J*=2.8 Hz, 6 H), 2.15 (br s, 3 H), 2.39 (s, 3 H), 3.49 (s, 3 H), 4.39 ppm (s, 2 H); LC/MS (ESI): *m/z* 342 [*M*+H]^+^; HRMS-ESI: *m/z* [*M*+H]^+^ calcd for C_14_H_20_N_3_O_3_S_2_: 342.0946, found: 342.09449; HPLC: *t*_R_=1.6 min (99 %) in 10 % H_2_O/CH_3_CN.

***N*****-(5-{[2-(Adamantan-1-yl)-2-oxoethyl]sulfanyl}-1,3,4-thiadiazol-2-yl)acetamide (50)**: To a solution of **48** (225 mg, 0.73 mmol) in AcOH (2 mL) was added Ac_2_O (0.1 mL). The mixture was stirred at RT overnight, then diluted with water (3 mL). The precipitate was collected, washed with water and dried in vacuo to give **50** as a white solid (228 mg, 89 %): mp: 208–210 °C; ^1^H NMR (270 mhz, CDCl_3_); *δ*=1.66–1.82 (m, 6 H), 1.81 (d, *J*=2.7 Hz, 6 H), 2.12 (br s, 3 H), 2.41 (s, 3 H), 4.38 ppm (s, 2 H); LC/MS (ESI): *m/z* 352 [*M*+H]^+^; HRMS-ESI: *m/z* [*M*+H]^+^ calcd for C_16_H_22_N_3_O_2_S_2_: 352.1153, found: 352.1131; HPLC: *t*_R_=1.9 min (99 %) in 10 % H_2_O/CH_3_CN.

***N*****-(5-{[2-(Adamantan-1-yl)-2-oxoethane]sulfinyl}-1,3,4-thiadiazol-2-yl)acetamide (51)**: Prepared using method C. A white solid (89 mg, 50 %): mp: 198–201 °C; ^1^H NMR (270 mhz, CDCl_3_): *δ*=1.65–1.85 (m, 12 H), 2.06 (br s, 3 H), 2.45 (s, 3 H), 4.36 (d, *J*=16 Hz, 1 H), 4.48 ppm (d, *J*=16 Hz, 1 H); LC/MS (ESI): *m/z* 368 [*M*+H]^+^; HRMS-ESI: *m/z* [*M*+H]^+^ calcd for C_16_H_22_N_3_O_3_S_2_: 368.1102, found: 368.1087; HPLC: *t*_R_=1.6 min (98 %) in 10 % H_2_O/CH_3_CN. ***N*****-(5-{[2-(Adamantan-1-yl)-2-oxoethane]sulfonyl}-1,3,4-thiadiazol-2-yl)acetamide (52)**: Prepared using method C. A white solid (20 mg, 11 %): mp: 216–218 °C; ^1^H NMR (270 mhz, CDCl_3_): *δ*=1.62–1.80 (m, 12 H), 2.07 (br s, 3 H), 2.47 (s, 3 H), 4.67 ppm (s, 2 H); LC/MS (ESI): *m/z* 384 [*M*+H]^+^; HRMS-ESI: *m/z* [*M*+H]^+^ calcd for C_16_H_22_N_3_O_4_S_2_: 384.1051, found: 384.1087; HPLC: *t*_R_=1.7 min (99 %) in 10 % H_2_O/CH_3_CN.

**Sodium 1-(adamantan-1-yl)-2-(5-methyl-1,3,4-thiadiazole-2-sulfonyl)ethen-1-ol (53)**: A solution of **37** (68 mg, 0.2 mmol) in 2 % aq NaOH (0.4 mL, 0.2 mmol) was shaken for 10 min. The reaction was concentrated in vacuo to give a white solid (72 mg, 100 %): ^1^H NMR (270 mhz, [D_6_]DMSO): *δ*=1.55–1.66 (m, 12 H), 1.90 (br s, 3 H), 2.68 (s, 3 H), 4.61 ppm (s, 1 H); LC/MS (ESI): *m/z* 341 [parent *M*+H]^+^; HRMS-ESI: *m/z* [parent *M*+Na]^+^ calcd for C_15_H_20_N_2_O_3_S_2_Na: 363.0813, found: 363.0795; HPLC: *t*_R_=1.9 min (98 %) in 10 % H_2_O/CH_3_CN.
